# Addressing graft-versus-host disease in allogeneic cell-based immunotherapy for cancer

**DOI:** 10.1186/s40164-025-00654-3

**Published:** 2025-05-02

**Authors:** Zibai Lyu, Siyue Niu, Ying Fang, Yuning Chen, Yan-Ruide Li, Lili Yang

**Affiliations:** 1 Department of Microbiology, Immunology & Molecular Genetics, University of California, Los Angeles, CA 90095 USA; 2 Department of Bioengineering, University of California, Los Angeles, CA 90095 USA; 3 Molecular Biology Institute, University of California, Los Angeles, CA 90095 USA; 4 Eli and Edythe Broad Center of Regenerative Medicine and Stem Cell Research, University of California, Los Angeles, CA 90095 USA; 5 Jonsson Comprehensive Cancer Center, David Geffen School of Medicine, University of California, Los Angeles, CA 90095 USA; 6 Parker Institute for Cancer Immunotherapy, University of California, Los Angeles, CA 90095 USA; 7 Goodman-Luskin Microbiome Center, University of California, Los Angeles, CA 90095 USA

**Keywords:** Graft-versus-host disease (GvHD), Allogeneic cell-based immunotherapy, Cancer therapy, CAR-T cells, Genetic engineering, TRAC, TRBC, NK cells, NKT cells, Stem cell technology

## Abstract

Allogeneic cell-based immunotherapies, particularly CAR-T cell therapy, represent a significant advancement in cancer treatment, offering scalable and consistent alternatives to autologous therapies. However, their widespread use is limited by the risk of graft-versus-host disease (GvHD). This review provides a comprehensive overview of GvHD in the context of allogeneic cell-based cancer immunotherapy and evaluates current strategies to mitigate its effects. Key strategies include genetic engineering approaches such as T cell receptor (TCR) knockout (KO) and T cell receptor alpha constant (TRAC) CAR knock-in. Alternative immune cell types like natural killer (NK) cells and natural killer T (NKT) cells offer potential solutions due to their lower alloreactivity. Additionally, stem cell technology, utilizing induced pluripotent stem cells (iPSCs), enables standardized and scalable production of engineered CAR-T cells. Clinical trials evaluating these strategies, such as UCART19 and CTX110, demonstrate promising results in preventing GvHD while maintaining anti-tumor efficacy. The review also addresses manufacturing considerations for allogeneic cell products and the challenges in translating preclinical findings into clinical success. By addressing these challenges, allogeneic cell-based immunotherapy continues to advance, paving the way for more accessible, scalable, and effective cancer treatments.

## Introduction

Cell-based therapy such as chimeric antigen receptor (CAR) T-cell therapy has transformed cancer treatment, especially for B-cell malignancies, with seven FDA-approved autologous CAR-T therapies demonstrating high response rates [1, 2]. These therapies have provided significant clinical benefits for patients with relapsed or refractory B-cell lymphoma, acute lymphoblastic leukemia, and multiple myeloma [2, 3]. However, despite their success, autologous CAR-T therapies face substantial limitations. The patient-specific manufacturing process is costly, time-intensive, and highly variable, leading to disparities in treatment efficacy [4, 5]. Patient-to-patient variability—arising from prior treatments and disease history—can impact cell expansion and function, contributing to a 2–10% production failure rate [6]. Furthermore, logistical challenges, including delays in cell production and delivery, can be particularly problematic for patients with rapidly progressing disease [4].

To overcome these obstacles, allogeneic or “off-the-shelf” CAR-T therapies have been developed as a promising alternative. Derived from healthy donors, these therapies offer scalability, reduced production time, and enhanced product consistency while eliminating the need for individualized manufacturing [4, 7, 8]. Additionally, allogeneic CAR-T therapies have the potential to lower costs and expand access to a broader patient population. However, a major barrier to their widespread clinical use is the risk of GvHD, a severe immune complication in which donor T cells attack the recipient’s healthy tissues [8]. Acute GvHD primarily affects the skin, gastrointestinal tract, and liver, occurring in 50–80% of cases, and can also impact the lungs, kidneys, eyes, and hematopoietic system, diminishing immune responsiveness [9–11]. Chronic GvHD, which develops later, involves multiple organs and shares characteristics with systemic autoimmune diseases, leading to long-term morbidity [12]. Due to these risks, allogeneic hematopoietic transplantation is generally limited to younger patients with good overall health, as the likelihood of regimen-related toxicity and GvHD increases with age [13].

To mitigate GvHD, several strategies are being explored. For instance, genetic engineering approaches, such as TCR KO via CRISPR/Cas9 or transcription activator-like effector nucleases (TALENs), can prevent alloreactive responses while maintaining therapeutic potency [14–17]. Additionally, alternative immune cell types with lower alloreactivity, such as NK cells or innate-like T cells, offer potential solutions [18–22]. These approaches aim to maximize the therapeutic benefits of allogeneic CAR-T therapy while minimizing immune complications.

This review provides a comprehensive analysis of the immunological mechanisms underlying GvHD in allogeneic cell-based cancer immunotherapy and evaluates current strategies for mitigating its effects. We examine clinical data from allogeneic CAR-engineered cell trials, assessing efficacy, safety, and feasibility. Additionally, we explore innovative approaches designed to enhance the effectiveness of allogeneic CAR-T therapy while reducing the risks associated with GvHD. By addressing these challenges, allogeneic cell-based immunotherapy continues to advance, paving the way for more accessible, scalable, and effective cancer treatments.

## Immunological basis of gvhd in allogeneic cell-based therapies

**Fig. 1:**
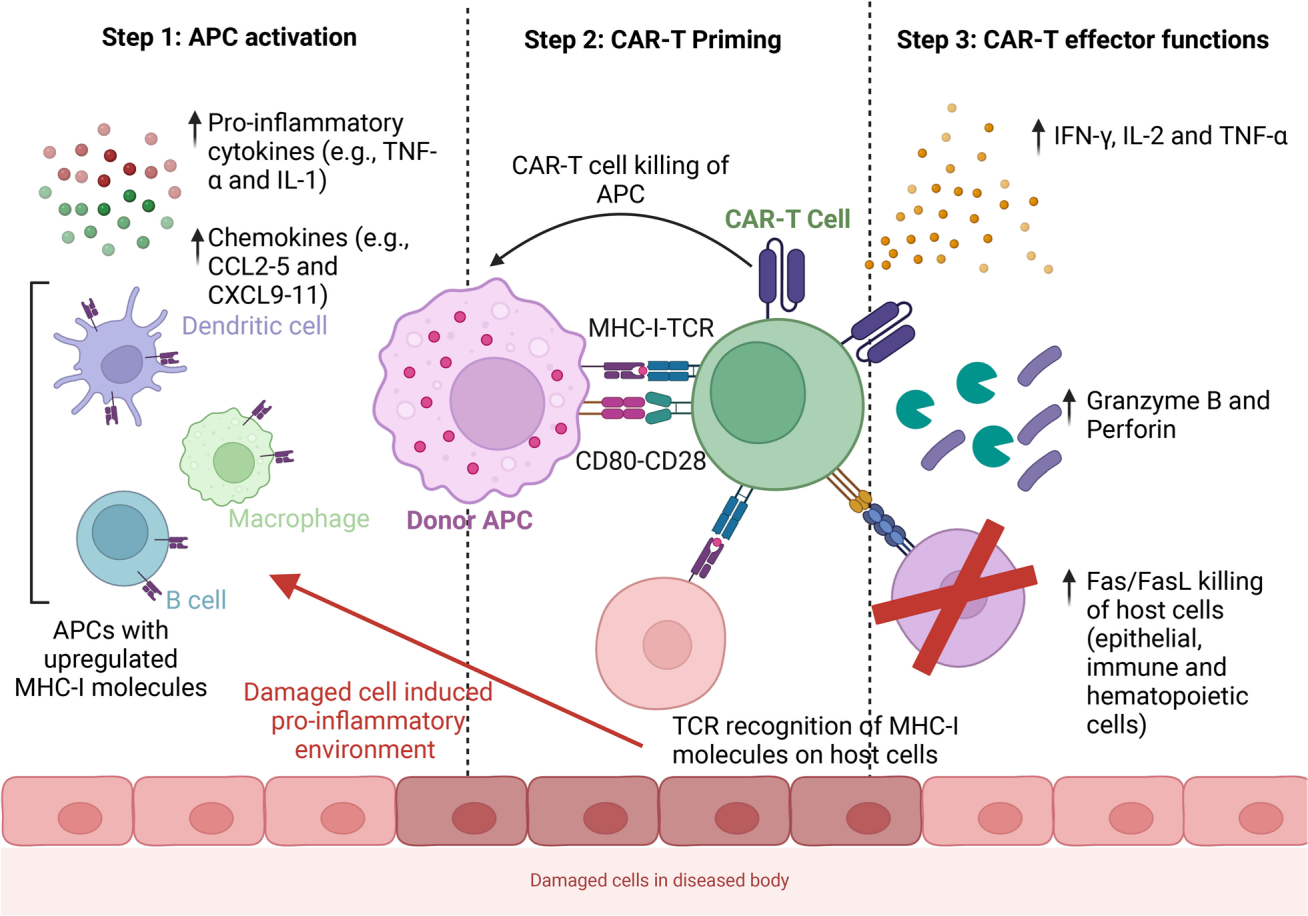
Immunological Basis of GvHD in Allogeneic CAR-Engineered Cell Therapies. Schematic representation of the immunological steps leading to CAR-T cell-mediated GvHD. Step 1: APC activation—Pro-inflammatory cytokines (e.g., TNF-α, IL-1) and chemokines (e.g., CCL2-5, CXCL9-11) upregulate MHC-I on APCs. Step 2: CAR-T priming—CAR-T cells recognize MHC-I on donor APCs, leading to activation. Step 3: CAR-T effector functions—Activated CAR-T cells release cytokines and mediate cytotoxicity via granzyme B/perforin and Fas/FasL, driving host cell apoptosis and GvHD pathology. APC, antigen-presenting cells; TNF- α, tumor necrosis factor alpha; IL-1, 2, 4, 5, 10, 13, interleukin 1, 2, 4, 5, 10, 13; CCL 2–5, chemokine (C–C motif) ligand 2–5; CXCL9-11, chemokine (C-X-C motif) ligand 9–11; IFN-γ, interferon gamma

GvHD progresses through a series of immunological events, beginning with the establishment of a pro-inflammatory environment, followed by human leukocyte antigen (HLA) antigen presentation, alloreactive T cell recognition, and ultimately, tissue damage mediated by inflammatory responses (Fig. 1).

Patients requiring cell-based therapies are typically in advanced disease stages, characterized by systemic stress and elevated levels of pro-inflammatory cytokines such as tumor necrosis factor (TNF)-α, interleukin (IL)-1, and chemokines including CCL2-5 and CXCL9-11 [23]. This inflammatory milieu enhances the activation of antigen-presenting cells (APCs) and upregulates the expression of major histocompatibility complex (MHC) antigens and co-stimulatory molecules on host APCs. Consequently, upon infusion, donor-derived T cells interact with host APCs and other cells that present mismatching MHC molecules, either recognizing self-antigens bound to MHC molecules or directly targeting foreign MHC molecules expressed by the host [24].

Upon initial antigen recognition, TCR signaling induces a conformational shift in adhesion molecules, enhancing binding affinity to APCs. This interaction facilitates full T cell activation through co-stimulatory molecules, leading to downstream immune responses. Fully activated T cells contribute to tissue damage via both cytokine secretion and direct cytotoxicity. Proinflammatory cytokines, including IFN-γ, IL-2, TNF-α, further amplify immune activation, exacerbating GvHD symptoms [23]. TNF-α enhances alloantigen presentation by APCs and induces direct tissue injury via apoptosis and necrosis [23, 25, 26]. IFN-γ promotes upregulation of chemokine receptors, MHC molecules, and adhesion proteins, while sensitizing monocytes and macrophages, rendering them hyperresponsive to secondary stimuli such as lipopolysaccharides (LPS) or infected target cells [27, 28].

The hallmark tissue tropism of GvHD, manifesting predominantly in the gastrointestinal (GI) tract, skin, and liver, can be attributed to the heightened immune sensitivity of these organs due to continuous antigen exposure [29, 30]. In addition to cytokine-mediated effects, CD8⁺ cytotoxic T cells from the donor contribute to direct host cell apoptosis via the Fas/Fas ligand (FasL) and perforin/granzyme pathways [31]. Specifically, Fas ligand on cytotoxic T cells binds to Fas receptors on target cells, triggering caspase-dependent apoptosis [32]. Alternatively, the perforin/granzyme axis facilitates membrane perforation, allowing granzymes to enter and activate caspase cascades, thereby inducing cell death [33].

The immunological landscape of autologous and allogeneic CAR-engineered cell therapies differs significantly due to their origins, persistence, and immune interactions within the host. Autologous CAR-T cells, derived from the patient’s own T cells, exhibit minimal risk of GvHD as they retain self-tolerance. However, their efficacy is often hindered by T cell exhaustion, especially in patients with heavily pretreated malignancies, where the endogenous T cell repertoire may be compromised. Additionally, autologous CAR-T cells must overcome the immunosuppressive tumor microenvironment (TME), characterized by TGF-β, IL-10, and immune checkpoint signaling (e.g., PD-1/PD-L1), which can attenuate CAR-T function [34]. The persistence of autologous CAR-T cells depends on intrinsic memory T cell formation and metabolic fitness, influencing long-term therapeutic outcomes.

In contrast, allogeneic CAR-T cells, derived from healthy donors, offer off-the-shelf accessibility, allowing for rapid administration without the delays associated with autologous cell manufacturing. However, allogeneic CAR-T therapies carry a heightened risk of GvHD, as donor-derived T cells can recognize host MHC molecules as foreign, leading to alloreactive T cell activation. This necessitates genome editing approaches, such as TCR KO or HLA silencing, to mitigate alloreactivity and prevent adverse immune responses. Moreover, allogeneic CAR-T cells may elicit host-versus-graft (HvG) responses, where the recipient's immune system targets infused cells for elimination, thereby reducing their persistence and therapeutic efficacy.

Overall, while autologous CAR-T therapies prioritize personalized, long-lasting responses, their success is limited by T cell exhaustion and immune evasion. Conversely, allogeneic CAR-T therapies offer scalable solutions but necessitate immune engineering to circumvent GvHD and host immune rejection, underscoring the need for advanced gene-editing and immunomodulatory strategies.

## In vitro and in vivo assays to evaluate gvhd

### In vitro assays

**Fig. 2:**
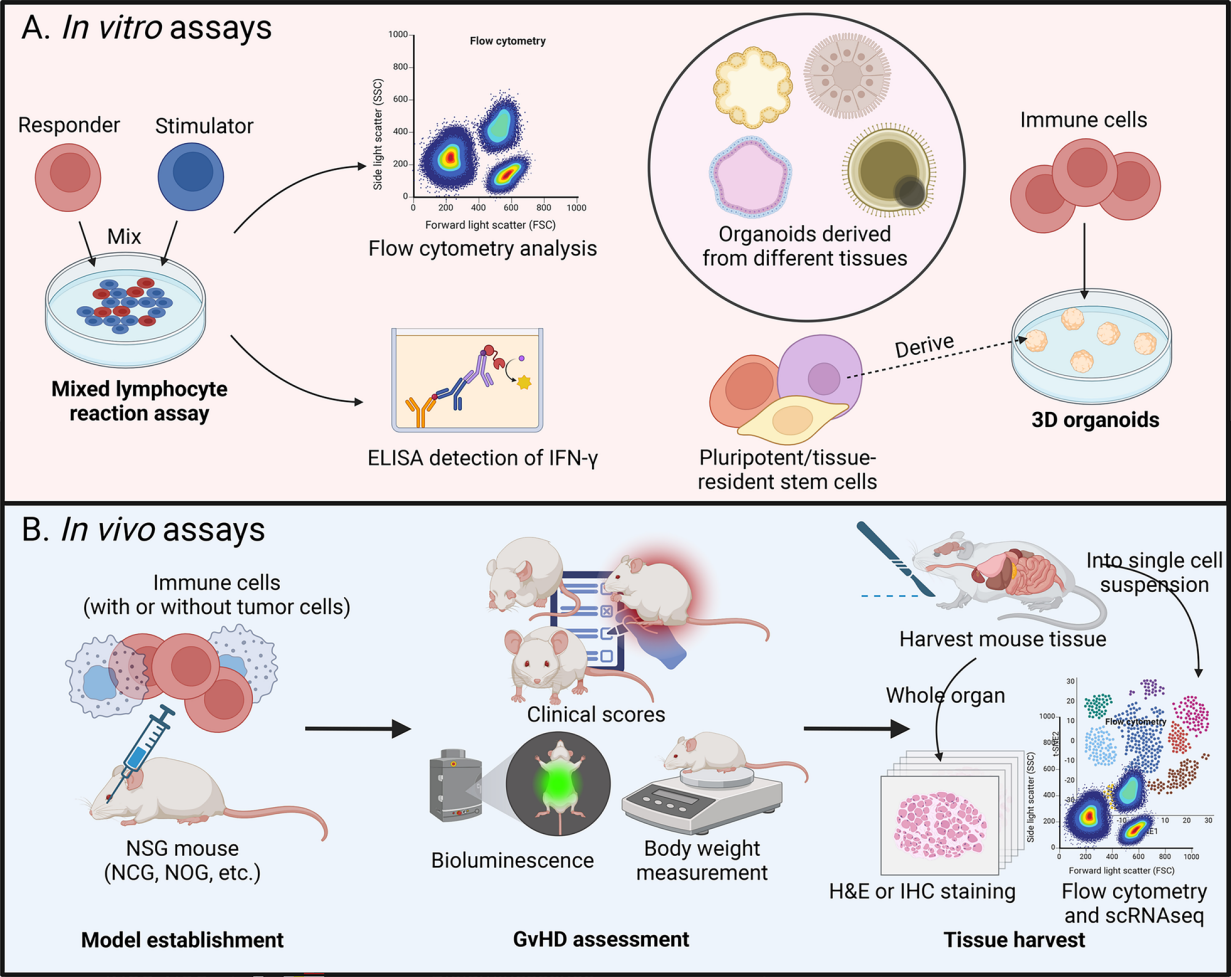
In Vitro and In Vivo Assays to Evaluate GvHD. Schematic representation of in vitro and in vivo assays used to evaluate GvHD in preclinical studies. The figure details the setup and analysis of **A** mixed lymphocyte assays and 3D organoid-immune cell coculture assays in vitro, as well as **B** the humanized mouse model in vivo. NSG, NOD/SCID/IL2Rγ^null^; NCG, NOD.Cg-Prkdc^scid^Il2rg^tm1Sug^; NCG, NOD/ShiLtJGpt-Prkdc^em26Cd52^Il2rg^em26Cd22^/Gpt; GvHD, graft-versus-host disease; H&E, hematoxylin and eosin; IHC, immunohistochemistry

To address GvHD associated with allogeneic CAR-engineered cells, various in vitro strategies have been implemented to evaluate the safety of allogeneic cell products. One way to measure GvHD related to allogeneic CAR-engineered cell therapies is the mixed lymphocyte reaction (MLR) assay (Fig. 2). MLR evaluates the interaction between genetically distinct lymphocytes cultured together, leading to blast transformation as an indicator of immune reactivity [8, 35]. In this assay, two genetically different lymphocyte populations are mixed: one serving as the effector (E) population and the other, rendered incapable of blast transformation through gamma irradiation, acting as the stimulator (S) population [35, 36]. After incubation, the final co-culture product is analyzed using tools such as flow cytometry and enzyme-linked immunosorbent assay (ELISA) [37, 38]. For example, in a study testing the GvH reaction of allogeneic IL-15 enhanced BCMA-targeting CAR-engineered natural killer T (^Allo15^BCAR-NKT) cells, irradiated healthy donor peripheral blood mononuclear cells (PBMCs) served as stimulators, while ^Allo15^BCAR-NKT cells were used as effectors. The co-cultured cells were subsequently analyzed for pro-inflammatory cytokine interferon-gamma (IFNγ) expression by ELISA [8, 39]. In another study investigating allogeneic CAR-T cells with of HLA-A/B and TRAC disruption against B cell malignancies, flow cytometry was performed after MLR assay to evaluate T cell activation and differentiation [38].

In addition to the current approaches mentioned above, organoid and three-dimensional (3D) tissue culture models have emerged as other potential tools in the study of GvHD (Fig. 2). Organoids are tissue-engineered, cell-based in vitro models that mimic key structural and functional characteristics of their corresponding in vivo tissues [40]. Derived from pluripotent or tissue-resident stem (embryonic or adult) cells, progenitor cells, or differentiated cells from healthy or diseased tissues, organoids retain many advantages of stem cells such as differentiating into assorted cell types, making them better substitutes for the native organs in evaluating GvHD. Matsuzawa-Ishimoto et al. developed an intestinal organoid platform to demonstrate the protective effect of ATG16L1 against GvHD following allogeneic cell transplantation [41]. Moreover, human intestinal and colonic crypts or purified stem cells can be embedded in Matrigel for improved organoid formation, which helps simulate the in vivo GvHD microenvironment [42–46]. For example, intestinal epithelial growth is promoted when laminin-rich Matrigel is used to mimic the crypt base [42].

In vitro assays, such as the MLR, provide a rapid and controlled approach to assess alloreactivity, making them valuable for screening GvHD risk in allogeneic CAR-engineered cells. These assays enable precise quantification of cytokine release and immune activation but lack the complexity of a full immune system. Emerging 3D organoid models improve tissue mimicry and facilitate the study of host-tissue interactions, yet they fail to capture systemic immune responses like cell trafficking and long-term inflammation. While in vitro models are essential for preliminary safety evaluations, their predictive value remains incomplete, necessitating validation through in vivo studies.

### In vivo models

Preclinical in vivo mouse models play an essential role in investigating GvHD pathogenesis, therapeutic cell behavior, and treatment efficacy in the context of allogeneic CAR-engineered cell therapy. These models provide a framework for systematically evaluating the safety and therapeutic potential of cell-based therapies. A foundational protocol established by Li et al. includes three key stages: model establishment, GvHD assessment, and tissue harvesting, each supported by specific experimental tools and approaches to enhance translational relevance [8, 47].

NOD/SCID/IL2Rγ^null^ (NSG) mice are foundational for xenogeneic GvHD modeling due to their lack of functional T, B, and NK cells, allowing efficient engraftment of human immune cells [48–50]. Researchers often inject human immune cells into these mice to establish a human-like immune system, creating a xenograft platform for studying allogeneic CAR-engineered cell therapies (Fig. 2). In some cases, such as allogeneic CAR-T therapy, this step can be omitted because human CAR-T TCRs can recognize murine MHC, inherently inducing xenogeneic GvHD-like pathology. To better replicate clinical conditions, tumor cells may also be co-infused.

Among NSG strains, the widely used NOD.Cg-Prkdc^scid^Il2rg^tm1Wjl^/Szj strain supports robust human immune cell engraftment, while the NOD.Cg-Prkdc^scid^Il2rg^tm1Sug^ (NOG) strain is particularly sensitive to xenogeneic GvHD, facilitating studies of immune-related pathologies [51]. The NOD/ShiLtJGpt-Prkdc^em26Cd52^Il2rg^em26Cd22^/Gpt (NCG) strain has been applied in B-cell acute lymphoblastic leukemia xenograft models to evaluate therapies such as induced natural killer (iNK) cells and CD19 CAR-engineered universal iNK cells [52]. The NOD.Cg-Prkdc^scid^Il2rg^tm1Sug^ Tg (CMV-IL2/IL15) (hNOG-IL15) strain, which expresses human IL-15, enables robust human NK cell engraftment and is particularly valuable for assessing CAR-NK cell therapies [53]. These specialized NSG sub-strains improve translational relevance by supporting investigations into GvHD, cancer xenografts, and immune cell-based therapies. Following the establishment of these models, allogeneic CAR-engineered cells targeting specific antigens are infused, simulating therapeutic administration and providing crucial insights into the interplay between therapeutic cells, tumor targets, and the host immune response.

After establishing the mouse model, it is essential to closely monitor the mice for signs of GvHD. The evaluation of GvHD progression relies on comprehensive assessments that combine clinical scoring with advanced biomarker analyses. Clinical scoring quantifies symptoms such as weight loss, activity changes, appearance and morphology, and organ-specific damage, providing a direct measure of GvHD severity (Fig. 2) [47]. Bioluminescence imaging (BLI) enables in vivo tracking of therapeutic cell migration and expansion, while cytokine profiling offers insights into systemic immune activation and cytokine release syndrome (CRS).

In a study using xenogeneic GvHD NSG mice, CAR-modified conventional T cells (CAR19-Tconvs) resulted in elevated levels of inflammatory cytokines, particularly IFNγ and TNFα, compared to control groups and CAR-modified double-negative T cells (CAR19-DNTs) [54]. This suggests a stronger inflammatory response in mice that developed GvHD. CAR19-Tconvs also exhibited higher intracellular levels of IFNγ and TNFα, whereas CAR19-DNTs expressed more modest levels in the absence of B-cell leukemia targets [54]. However, when leukemia targets were present, both CAR19-DNTs and CAR19-Tconvs showed elevated levels of these cytokines. Similarly, co-culture with NALM-6 or Daudi cells triggered higher IFNγ release from both CAR19-DNTs and CAR19-Tconvs compared to NT-DNTs.

In another study using hIL15-NOG mice, CAR.CD123-NK cells exhibited significantly higher plasma concentrations of Granzyme B and IFNγ by day 30 post-infusion, indicating enhanced anti-leukemia activity [53]. This study also revealed that the hIL15-NOG model enabled the detection of inflammatory cytokines in plasma, a feature not observed in the NSG model. Furthermore, clinical observations in patients showed modest elevations of IL-6 and IL-1β following CAR-NK cell infusion, reflecting a milder inflammatory profile compared to the severe cytokine release observed in some CAR-T therapies [55].

Tissue harvesting and analysis are crucial for characterizing immune cell infiltration and tissue damage associated with GvHD. When severe symptoms develop and euthanasia is required for the established mice model, tissues from primary GvHD target organs, including the skin, liver, and gastrointestinal tract, are collected for histological analysis, such as H&E and IHC staining, to assess immune cell infiltration, tissue destruction, and fibrosis (Fig. 2) [47, 56]. Flow cytometry of single-cell suspensions provides a detailed view of immune cell populations involved in GvHD, including CD4⁺ and CD8⁺ T cells, regulatory T cells (Tregs), and other immune subsets.

Patterns of immune infiltration can reveal therapy-specific differences in safety and efficacy. For instance, CAR19-Tconv-treated mice exhibit significant T cell infiltration and tissue damage, indicative of severe GvHD. In contrast, CAR19-DNT-treated mice show minimal tissue infiltration and histological changes, highlighting their reduced toxicity. In studies using CAR.CD123-T cells, robust immune cell infiltration is observed in the injured vasculature, whereas CAR.CD123-NK cells show no off-target activity, indicating their potential for safer therapeutic applications [53, 54].

Together, NSG-based xenograft models play a crucial role in assessing the in vivo behavior of allogeneic CAR-engineered cells, including their persistence, expansion, and potential to induce GvHD. These models enable dynamic monitoring of disease progression through clinical scoring, cytokine profiling, and histopathological evaluation of affected organs. Specialized NSG sub-strains further refine these studies by facilitating the engraftment of specific immune subsets, such as CAR-NK cells in hIL15-NOG mice. However, xenogeneic GvHD in NSG mice is often more severe than clinical GvHD due to species-specific differences in immune regulation, potentially leading to an overestimation of risk. Additionally, these models lack fully functional adaptive immunity, limiting their ability to fully replicate human immune responses. Despite these limitations, in vivo models remain indispensable for translational research, particularly when complemented with clinical biomarker analyses to enhance predictive accuracy.

## Strategies to address GVHD

### TCR Knockout

**Fig. 3:**
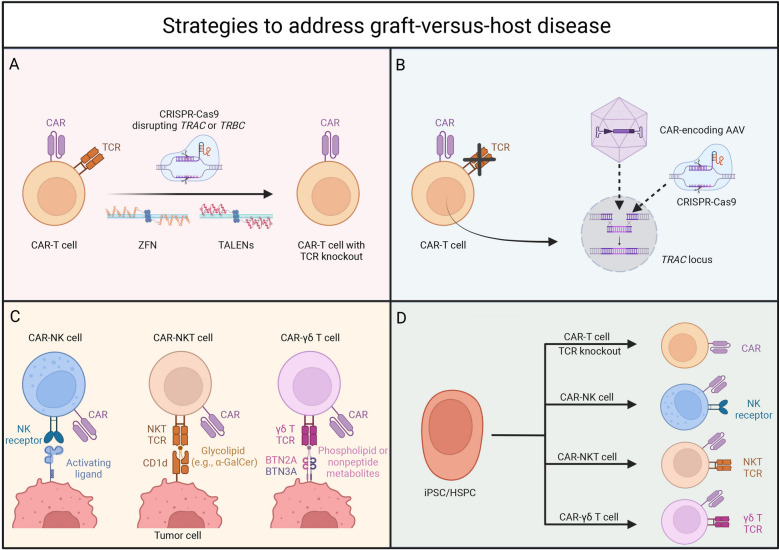
Strategies to Mitigate GvHD in Allogeneic CAR Therapies. **A** TCR knockout. Genome editing (CRISPR-Cas9, ZFNs, TALENs) disrupts *TRAC* or *TRBC* to prevent TCR-mediated alloreactivity. **B** TRAC CAR knock-in. The CAR transgene is inserted into the *TRAC* locus using CRISPR-Cas9 and AAV, ensuring uniform CAR expression while eliminating TCR signaling.** C**. Use of NK and innate-like T cells. CAR-engineered NK, NKT, and γδ T cells, which have low alloreactivity, provide alternative effectors to minimize GvHD risk.** D**. Stem cell technology. iPSCs or HSPCs generate CAR-T cells with TCR knockout, CAR-NK, CAR-NKT, and CAR-γδ T cells, offering a scalable, off-the-shelf solution with reduced GvHD potential. AAV, adeno-associated virus; BTN, butyrophilin; CAR, chimeric antigen receptor; CRISPR, clustered regularly interspaced short palindromic repeats; GvHD, graft-versus-host disease; HSPC, hematopoietic stem and progenitor cell; NKT, natural killer T; iPSC, induced pluripotent stem cell; NK, natural killer; TALEN, transcription activator-like effector nuclease; TCR, T-cell receptor; ZFN, zinc finger nuclease

In allogeneic CAR-T cell therapy, donor-derived T cells expressing αβ TCRs can recognize foreign HLA molecules, leading to acute or chronic graft-versus-host disease GvHD [57]. To prevent this, gene-editing strategies targeting the TCR complex—specifically the T cell receptor alpha constant (*TRAC*) and T cell receptor beta constant (*TRBC*) genes—have been developed using CRISPR-Cas9, TALENs, and zinc finger nucleases (ZFNs) (Table 1; Fig. 3) [58–62]. Since only fully assembled TCR complexes are transported to the cell surface, knocking out *TRAC* or *TRBC* effectively eliminates αβ TCR expression, preventing GvHD. Notably, TRAC has only one constant region, making it a more straightforward target than *TRBC*, which has two (*TRBC1* and *TRBC2*) [63].

**Table 1:** Strategies to address GvHD

Strategy	Advantage	Disadvantage
TCR knockout	Clinically validated Multiple gene-editing options	Off-target effects
*TRAC* CAR knock-in	Controlled CAR insertion Preserved T cell function Precise gene editing	Inefficient in T cells due to HDR Challenges in large-scale use
Use of NK cells and other innate T cells	No MHC restriction Lower CRS/neurotoxicity risk	Limited persistence and expansion Standardization challenges
Stem cell technology	Unlimited engineered cells Precise modifications Scalable manufacturing	Complex protocols Potential tumorigenicity Variability of cell products

Preclinical and early-phase clinical trials have demonstrated the feasibility of TCR KO for allogeneic CAR-T therapy. Torikai et al., first explored TCR disruption in CAR-T cells, using ZFNs to knock out *TRAC* or a consensus sequence shared by *TRBC1* and *TRBC2* in CD19-targeting CAR-T cells [60]. Their results showed that eliminating either *TRAC* or *TRBC* prevented TCR expression while maintaining CAR specificity against CD19, making allogeneic CAR-T therapy viable [60]. Following that, Sommer et al. utilized TALENs to disrupt *TRAC*, successfully preventing xenogeneic GvHD while preserving T cell functionality and antitumor activity *in vivo* [64]. Utilizing CRISPR-Cas9, Cooper et al. also focused on *TRAC* disruption in CAR-T cells targeting human T-cell acute lymphoblastic leukemia (T-ALL) and showed efficacy without inducing xenogeneic GvHD, making allogeneic T cell therapy for relapsed and refractory T-ALL and non-Hodgkin’s T cell lymphoma treatment possible [65].

In clinical settings, UCART19, the first allogeneic CD19-targeting CAR-T cell therapy incorporating TRAC disruption via TALENs, reduced GvHD incidence to grade 1 skin GvHD in 10% and 8% of patients in 2020 and 2022, respectively [66]. Other trials, including ALLO-501 (CD19-targeting), ALLO-715 (BCMA-targeting), and UCART22 (CD22-targeting), also employed TALEN-mediated TRAC disruption and reported no GvHD cases [67–69]. Similarly, CRISPR-Cas9-engineered allogeneic CAR-T therapies, such as CTX110 (CD19), CTA101 (CD19/CD20), WU-CART007 (CD7), and TyU19 (CD19), showed no GvHD occurrence [70–73]. Notably, P-BCMA-ALL01, the only reported trial involving *TRBC* KO, used the Cas-CLOVER™ gene-editing system and also reported no GvHD [74]. Together, these pre-clinical studies and reported clinical trials also showed the necessity and effectiveness of TCR disruption in the development of allogeneic CAR-T cell products.

### TRAC car knock-in

Beyond simply knocking out *TRAC* or *TRBC* to disrupt the TCR, another strategy involves combining TCR deletion with CAR transgene integration using adeno-associated virus (AAV)-based vectors (Table 1; Fig. 3) [17, 75]. This approach enables precise CAR insertion into the *TRAC* gene locus, thereby avoiding the random integration of CAR genes that typically occurs with lentiviral vectors [75–78]. Integration is completed through homology-directed recombination (HDR) following *TRAC* disruption by nucleases such as Cas9, *TRAC* megaTAL, or TRC1-2 (a single-chain variant of I-CreI) [78]. MacLeod et al. engineered a site-specific endonuclease, TRC1-2, to induce DNA breaks with high frequency at the *TRAC* locus, effectively knocking out TCR expression [78]. They transduced cells with an AAV6 vector carrying an anti-CD19-BB-zeta CAR expression cassette flanked by *TRAC* homology arms, achieving highly efficient CAR integration into the *TRAC* locus [78]. Their gene-edited, TCR-deficient anti-CD19 CAR-T cells demonstrated potent in vitro and in vivo responses against CD19-expressing tumor cells [78]. Similarly, Hale et al. employed the *TRAC* megaTAL nuclease to insert CAR constructs into the *TRAC* locus via HDR, generating TCR-negative anti-CD19 and anti-BCMA CAR-T cells [77]. These engineered T cells exhibited robust cytolytic activity against both tumor cell lines and primary tumor cells Tables 2, 3.

**Table 2:** Reported clinical trials of allogeneic cell products with endogenous TCR disruption

Methods	Rationale	Gene editing tools	Clinical trial examples	Clinical results regarding GvHD	Clinical results regarding other safety issues	Clinical results regarding efficacy	Reference and NCT number
Disruption of TRAC	Elimination of endogenous TCR expression by knocking out TRAC	TALENs	UCART19, allogeneic CD19-targeting CAR-T (2020)	Grade 1 acute skin GvHD was observed in 10%	Grade 1–2 CRS was reported in 91% of patients; grade 3–4 CRS was reported in 14%; grade 1 or 2 neurotoxicity was reported in 38%; grade 4 prolonged cytopenia was reported in 32%; two treatment-related deaths were reported due to neutropenic sepsis and pulmonary hemorrhage	Infused CAR-T cells were reported to expand and persist for a median duration of 4.1 months in 71% of patients, and CR was reported in 67% of patients	NCT02808442 and NCT02746952 [66]
			ALLO-501, allogeneic CD19-targeting CAR-T (2021)	No GvHD was observed	No dose-limiting toxicities (DLT) or immune effector cell-associated neurotoxicity syndrome (ICANS) were reported; grade 1–2 CRS was reported in 21.7%; cytopenia was reported in 82.6%; grade 3 + infections were reported in 23.9%; 5 patients died after treatment	ORR was 75%; CR was 50%; the longest observed ongoing CR was over 15 months	NCT03939026 [67]
			ALLO-501A, allogeneic CD19-targeting CAR-T (2021)	No GvHD was observed	No CRS, ICANS, DLT, or grade 3 + infections were observed; cytopenia was reported in 72% of patients	ORR was 50%; CR was reported in 50%; in the consolidation group, both ORR and CR were 66.7%	NCT04416984 [138]
			UCART19, allogeneic CD19-targeting CAR-T (2022)	Grade 1 acute skin GvHD was reported in 8% patients	DLTs were reported in 12% of patients; grade 3 + CRS was reported in 24%; grade 4 prolonged cytopenia was reported in 8%; grade 3 + neurotoxicity was reported in 4%; grade 3 + infections were reported in 28%	ORR was 48%; median relapse-free survival was 7.4 months; progression-free survival was 2.1 months; overall survival was 13.4 months	NCT02746952 [139]
			ALLO-715, allogeneic BCMA-targeting CAR-T (2023)	No GvHD was reported	Grade 3 + adverse events were reported in 88.0% of patients; CRS was reported in 55.8%, with grade 3 + in 2.3%; neurotoxicity was reported in 14% with no grade 3 + ; infections were reported in 53.5%, with grade 3 + in 23.3%	Overall, 55.8% of patients responded; among patients treated with 320 × 10⁶ CAR-T cells, 70.8% responded, including 45.8% with very good partial response or better and 25% with complete/stringent complete responses; the median response duration was 8.3 months	NCT04093596 [68]
			UCART22, allogeneic CD22-targeting CAR-T (2023)	No GvHD was reported	No grade 3 + CRS, DLTs, or ICANS were reported	The response rate at DL2 was 67% and 50% at DL3	NCT04150497 [69]
		CRISPR-Cas9	CTX110, allogeneic CD19-targeting CAR-T (2022)	No GvHD was reported	CRS was reported in 56% of patients, ICANS was reported in 9.4%; grade 3 + infections was reported in 12.5%	Best ORR and CR rates were 67% and 41%; 6-month CR rate was 19%	NCT04035434 [70]
			CTA101, allogeneic CD19/CD20 dual-targeting CAR-T (2022)	No GvHD was reported	Grade 1–2 CRS was reported in 83.3% of patients, grade 3 CRS was reported in 16.7%; no DLTs or neurotoxicity were reported; grade 3 + infections were reported in 50%; cytopenia was reported in 50%	CR was 83.3%; at a median follow-up of 4.3 months, 60% patients with CR/CR with incomplete hematologic recovery (CRi) remained minimal residual disease (MRD)-negative	NCT04227015 [71]
			WU-CART007, allogeneic CD7-targeting CAR-T (2023)	No GvHD was reported	CRS was reported in 67% of patients; grade 1 ICANS was reported in 8.3%; no prolonged T cell aplasia or pancytopenia was reported	The ORR at ≥ dose level (DL) 2 of evaluable patients was 43%; with a median follow-up of 107 days, responses lasted up to 86 days	NCT04984356 [72]
			TyU19, allogeneic CD19-targeting CAR-T (2024)	No GvHD was reported	No CRS was reported	The infused CAR-T cells persisted for more than 3 months, reaching complete B-cell depletion within 2 weeks of treatment	NCT05859997 [73]
Disruption of TRBC	Elimination of endogenous TCR expression by knocking out TCR beta chain 1	Cas-CLOVER™ Site-Specific Gene Editing System	P-BCMA-ALL01, allogeneic BCMA-targeting CAR-T (2023)	No GvHD was reported	No DLT was reported; grade 1 CRS was reported in 14% of patients; grade 1 ICANS was reported in 4%; treatment emergent adverse events (TEAEs) reported were anemia (36%), neutropenia (36%), constipation (36%), and leukopenia (32%)	N/A	NCT04960579 [74]
TRAC CAR knock-in	Introduction of CAR into the TRAC locus to replace endogenous TCR expression	CRISPR-Cas9	CTX130, allogeneic CD70-targeting CAR-T (2024)	no GvHD was reported	No DLTs or ICANS were reported; grade 1–2 CRS was reported in 50% of patients; grade; no grade 3 + CRS was reported	Disease control was reported in 81.3% of patients	NCT04438083 [80]
			TT52CAR19, allogeneic CD19-targeting CAR-T (2024)	16.7% of patients developed skin GvHD	Grade II CRS was reported in 33.3% of patients., transient grade IV neurotoxicity was reported in 16.7%	Cell expansion and flow cytometric remission were reported in 66.7% of patients	NCT04557436 [79]

**Table 3:** Reported clinical trials of allogeneic cell products using alternative cell sources with low GvHD risk

Cell source	Rationale	Clinical trial examples	Clinical results regarding GvHD	Clinical results regarding other safety issues	Clinical results regarding efficacy	Reference and NCT number
Cord blood-derived NK cells	NK cells do not depend on HLA recognition for cytotoxicity	HLA-mismatched CD19-targeting CAR-NK (2020)	No GvHD was reported	No CRS or neurotoxicity was reported; inflammatory cytokine levels remained unchanged from baseline	Responses were reported in 73% of patients, with infused CAR-NK cells expanding and persisting at low levels for at least 12 months	NCT03056339 [83]
NKT cells isolated from the leukapheresis product of 1 HLA-unmatched healthy individual	Monomorphic CD1d-restricted NKTs do not exhibit alloreactivity	Allogeneic CD19-trageting CAR-NKT (2021)	No GvHD was reported	Grade 1 CRS was reported in 20% of patients	CR was reported in 1 of 2 ALL patients and 2 of 7 NHL patients; the therapeutic cells persisted up to 12 weeks post-infusion	NCT00840853 [18]

Clinically, TT52CAR19, an allogeneic CD19-targeting CAR-T, incorporating self-duplicating CRISPR guide RNA expression cassettes within the 3’ long terminal repeat of a CAR19 lentiviral vector, was tested in children with refractory B cell leukemia [79]. The trial reported a 16.7% incidence of skin GvHD, while CAR-T expansion in was observed in 66.7% of patients [79]. Another trial, CTX130, an allogeneic CD70-targeting CAR-T, utilized CRISPR-Cas9 gene editing to insert an anti-CD70 CAR expression cassette into the *TRAC* locus via HDR [80]. No cases of GvHD were reported, and disease control was achieved in 81.3% of patients [80]. These studies highlight the feasibility of simultaneously disrupting TCR expression and integrating CAR transgenes, offering a promising approach to enhancing the safety and efficacy of allogeneic CAR-T cell therapies.

### Use of NK cells and other innate T cells

As evidenced above, allogeneic cell-based therapies with T cells as the primary cell types bear high risks of inducing severe GvHD without further modifications, which brought in uncertainties regarding safety and functions of T cells. Therefore, it is essential to consider innate or innate like immune cells, which are not activated by heterogenies MHC presenting antigens. Specifically, NK cells, γδ T cells, NKT cells, and other innate lymphocytes are promising candidates for CAR-engineered cell therapy.

NK cells, as effector lymphocytes of the innate immune system, play a pivotal role in tumor surveillance, with activation governed by a dynamic interplay of activating and inhibitory surface receptors. Stress-induced ligands, such as ULBP1, CD155, and CD112, are frequently upregulated in tumor cells and are recognized by NK cell receptors like NKG2D, NKp30, and NKp44, thereby providing an additional anti-tumor mechanism [81]. Conversely, NK cells are inhibited by self-MHC class I molecules meaning, the loss of MHC class I, a hallmark of many tumor cells, triggers NK cell activation [81]. Unlike T cells, NK cells do not depend on antigen recognition via MHC but instead leverage innate recognition patterns, allowing them to circumvent antigen escape mechanisms and persist in eradicating residual tumor cells following prolonged treatment without triggering GvHD [82].

This intrinsic targeting mechanism through NKRs, independent of CAR and MHC-mediated recognition, minimizes the risk of antigen escape and renders NK cells non-reactive towards HLA-mismatched cells, significantly reducing the risk of GvHD. Furthermore, NK cells are less likely to induce cytokine release syndrome or neurotoxicity due to their controlled cytokine release and limited proliferation compared to T cells. Specifically, NK cells suppress the production of pro-inflammatory cytokines such as TNF-α, IL-1, and IL-6, which are typically elevated in CAR-T cell therapies [83, 84]. However, these properties also present challenges for CAR-NK cell therapies, including reduced persistence and limited long-term efficacy where it is difficult to maintain large-scale production as they are challenging to expand and preserve, limited in vivo survival without exogenous cytokine support, and susceptibility to trogocytosis-mediated self-damage [85–87].

Despite these challenges, CAR-NK cells have shown promise in clinical studies targeting hematologic malignancies and solid tumors such as ovarian cancer, endometrial cancer, and glioblastoma [88, 89]. Future advancements in optimizing NK cell survival, expansion, and functionality may unlock their potential as allogeneic cell therapies for cancer.

γδ T cells have emerged as a compelling focus in immunotherapy due to their unique structural and functional attributes, which distinguish them from conventional αβ T cells. Unlike αβ T cells, γδ T cells express a distinct TCR architecture composed of γ and δ chains, enabling the recognition of a diverse array of antigens in an MHC-independent manner. This capability allows γδ T cells to maintain effective tumor-killing activity even in the face of antigen escape, while their ability to bypass MHC-mediated antigen presentation significantly reduces the risk of GvHD [90–92]. Furthermore, while αβ T cells rely on dual signals—antigen recognition and co-stimulatory input—for activation, γδ T cells can respond robustly to a single activating signal, highlighting their functional versatility [93]. Adding to this diversity, certain subsets, such as Vδ2 T cells, act as professional antigen-presenting cells, further broadening the immunological roles γδ T cells can fulfill.

These distinctive properties have fueled growing interest in CAR γδ T cells as a therapeutic strategy for various malignancies, including B-cell lymphoma, glioblastoma, and colorectal cancer, with ongoing clinical trials evaluating their efficacy and safety [94]. Despite their promise, several challenges must be addressed to unlock the full therapeutic potential of CAR γδ T cells. A significant obstacle lies in the extensive heterogeneity of γδ T cell subsets, which display diverse antigen recognition patterns, tissue homing properties, and effector functions, complicating the standardization of genetic engineering approaches [95]. Additionally, the lack of optimal gene delivery methods and the reduced persistence of γδ T cells in the immunosuppressive tumor microenvironment further impede their clinical application [96, 97]. Overcoming these challenges will be crucial to advancing CAR γδ T cell therapies and harnessing their potential across a range of oncological and clinical settings.

NKT cells are a subset of lipid- and glycolipid-reactive T lymphocytes that express NK cell markers such as NKp44 [98, 99]. Like NK cells and γδ T cells, NKT cells do not rely on MHC molecules to recognize peptide antigens. Instead, they recognize endogenous, polymorphic, MHC-like CD1d molecules and rapidly release cytotoxic granules (e.g., granzyme B, perforin), apoptotic signals (e.g., FasL → Fas), and immunomodulatory cytokines, including IFNγ, TNF, and IL-4 [100]. This enables them to kill tumor cells and modulate downstream immune responses [101].

The combination of endogenous NKT TCRs recognizing CD1d, NKRs targeting NKR ligands, and CARs engineered to identify specific antigens equips CAR-NKT cells with a triple-targeting capability. This multi-pronged approach enhances their precision and efficacy, allowing them to target a broad range of environments, including scenarios of tumor antigen escape. Moreover, because CAR-NKT cells do not depend on MHC molecules and CD1d is polymorphic, the risk of GvHD is reduced. Additionally, similar to NK cells, NKT cells produce fewer pro-inflammatory cytokines, minimizing the likelihood of CRS or neurotoxicity.

Despite these advantages, CAR-NKT cell therapy faces several challenges. These include limited expansion and persistence in vivo, loss of viability after cryopreservation, and the potential induction of an anergic phenotype following α-GalCer stimulation, which diminishes therapeutic efficacy [102–106]. Furthermore, low infiltration and impaired function in solid tumors, combined with insufficient autologous NKT cell counts in patients, hinder the feasibility of autologous therapies, particularly for patients with advanced diseases. These challenges underscore the importance of developing allogeneic CAR-NKT cell therapies.

To further evaluate the therapeutic potential of CAR-NKT cells, ongoing clinical trials are investigating their efficacy in refractory neuroblastoma and B-cell malignancies [107, 108].

### Stem cell technology

iPSCs represent a versatile and ethical source for engineered cancer therapies. Derived from adult tissues like skin or blood, iPSCs can be expanded and differentiated into various cell types, providing an unlimited supply for therapeutic use [109]. iPSCs ensure product consistency by enabling uniform genetic modifications and large-scale manufacturing, which is very suitable for allogeneic cell products (Table 1; Fig. 3). iPSC’s susceptibility to gene editing, such as CRISPR-Cas9, TALENs, and lentiviral transduction, facilitates precise modifications to minimize GvHD risk [110].

The most direct method for reducing GvHD in iPSC-derived T cells involves disrupting the TCR genes. Gene editing techniques, such as disrupting the *TRAC* or *TRBC* genes, can eliminate the endogenous TCR, preventing GvHD [111]. Eyquem et al. used CRISPR/Cas9 to target the *TRAC* locus, integrating a CD19-specific CAR construct while knocking out the TCR and simultaneously introducing the CAR [17]. This method could also be applied to iPSCs for developing allogeneic CAR therapies. Furthermore, introducing a transgenic TCR into iPSCs prior to differentiation generates signals through the CD3 complex during T cell development, mimicking normal TCR signaling [112]. This inhibits the rearrangement of endogenous TCRs, ensuring that only the engineered TCR is expressed on the differentiated T cells. This approach enables the targeted recognition of specific antigens, enhancing the safety and effectiveness of these cells for therapeutic applications, as demonstrated in several preclinical studies [17, 78, 113, 114].

In addition to TCR modification, HLA editing can further reduce the risk of allorejection. By disrupting MHC-I and MHC-II molecules through gene editing tools targeting *B2M*, *CIITA*, or *RFX*, T cell-mediated host-versus-graft rejection can be minimized. However, removing MHC-I may increase susceptibility to NK cell-mediated rejection. To address this, NK-inhibitory ligands such as β2m-HLA-E or Siglec-7/9 can be introduced, and disruption of NK-activating ligands like CD155 further mitigates NK-mediated rejection. These strategies collectively enable the creation of “universal” CAR-T cells that are compatible with HLA-mismatched recipients [36].

Preclinical and clinical studies demonstrate the transformative potential of stem cell-derived allogeneic CAR therapies. iPSC-derived CAR-T cells exhibit potent antitumor activity in vitro and in vivo, lysing tumor cells and secreting tumor-lytic enzymes without the need for exogenous cytokines. For example, Wang et al. demonstrated that iPSC-derived CD19-CAR T cells, generated using a 3D-organoid culture system, showed potent antitumor efficacy in vivo [115]. Similarly, iPSC-derived CAR-NKT cells have shown prolonged tumor control, enhanced survival in xenograft models, and potent cytotoxicity against HER2-positive cancer cell lines in vitro [116]. CAR-γδ-T cells have displayed long-term tumor-killing capacity and eliminated engrafted tumor cells in vivo without cytokine support [117]. Notably, dual-antigen receptor-bearing T cells targeting CD19 and LMP2 achieved superior survival outcomes in tumor-inoculated mice compared to single-antigen-targeted cells [118]. Antigen-specific iPSC-derived CAR-T cells can employ both CAR and TCR mechanisms to eradicate malignant cells, while virus-specific iPSC-derived CAR-T cells have demonstrated the dual ability to prevent post-transplant infections and mediate anti-tumor cytotoxicity [118]. Additionally, iPSC-derived CD4 + CAR-Treg-like cells have effectively controlled GvHD in xenograft models, suppressing disease progression and inhibiting cytotoxic T cell proliferation, as evidenced by studies like those conducted by Yano et al. [119].

Clinically, these advancements are being tested in ongoing trials. Recently, Ghobadi et al. report a Phase 1 trial of FT596, an iPSC-derived CAR NK cell therapy for relapsed B-cell lymphoma, showing promising safety and efficacy results [120]. Another Phase I trial is investigating FT819, an iPSC-derived, TCR-less CD19-CAR T-cell therapy for relapsed/refractory B-cell lymphomas, chronic lymphocytic leukemia, and B-cell acute lymphoblastic leukemia [121]. iPSC-derived NKT cells are being evaluated in a Phase I trial for advanced head and neck cancer, while virus-specific CAR-T cells and other hPSC-based therapies are also under investigation (jRCT2033200116). Additionally, mesenchymal stem cells (MSCs) derived from iPSCs, such as Cymerus MSCs, are being explored for treating steroid-resistant acute GvHD. Early clinical studies suggest these MSCs may improve outcomes following allogeneic hematopoietic stem cell transplantation [122]. Together, these preclinical and clinical efforts underscore the potential of iPSC-derived therapies to revolutionize cell-based immunotherapies.

### Other approaches

Beyond altering the cellular composition of CAR-expressing cells to mitigate GvHD, regulatory immune cells offer a promising avenue for prophylaxis and treatment.

Tregs, a specialized subset of T cells, suppress effector T-cell responses through multiple mechanisms, including the secretion of immunosuppressive cytokines such as IL-10, IL-35, and transforming growth factor-beta (TGF-β). Additionally, Tregs compete for IL-2, a cytokine essential for effector T-cell survival, via high expression of the IL-2 receptor, thereby limiting effector T-cell expansion. They also modulate APC function by interfering with dendritic cell (DC) activation and maturation, reducing T-cell priming efficiency [123].Another key regulatory population, MSCs, exhibit immunomodulatory properties without eliciting immunogenic responses due to the absence of MHC-II expression. MSCs have demonstrated efficacy in clinical trials for GvHD, with patients exhibiting resolution of disease symptoms and complete responses (CRs) [124]. Their therapeutic effects are mediated through secretion of immunosuppressive factors, including indoleamine 2,3-dioxygenase (IDO), in response to IFN-γ and TNF-α, leading to inhibition of effector T-cell proliferation [125]. Furthermore, MSCs modulate the cytokine milieu, shifting pro-inflammatory T-helper 1 (Th1) responses toward an anti-inflammatory Th2 profile, suppressing DC and NK cell activation, and facilitating tissue repair [124, 126].

In addition to cell-based therapies, small molecules remain a mainstay in GvHD management. Calcineurin inhibitors (CNIs), including cyclosporine and tacrolimus, serve as the cornerstone of GvHD prophylaxis following allogeneic hematopoietic cell transplantation and can be adapted to other allogeneic cell therapies. CNIs prevent GvHD by blocking T-cell activation via inhibition of calcineurin-dependent nuclear factor of activated T cells (NFAT) signaling and suppression of Lck-S59 dephosphorylation, both critical pathways in acute GvHD [127]. However, their broad immunosuppressive activity extends beyond pathogenic T cells, potentially reducing the efficacy of adoptive cellular therapies. Notably, tacrolimus has been shown to directly inhibit CAR-T cytotoxicity in vitro, reducing tumor cell killing by over 50% [128]. Additionally, CNIs preserve alloreactive CD4 + central memory T cells, increasing the risk of chronic GvHD and are associated with significant toxicities, including nephrotoxicity and hypertension, with intolerance reported in up to 20% of pediatric patients [129–131].

Alternative small-molecule approaches are emerging to address these limitations. Post-transplant cyclophosphamide (PTCy) preferentially targets rapidly dividing T cells through induction of DNA damage via alkylating metabolites, reducing alloreactivity while sparing regulatory immune subsets [132]. Abatacept-based regimens, which block CD28-mediated T-cell co-stimulation through competitive binding to CD80/CD86 on APCs, have shown efficacy in preventing T-cell activation [133, 134]. Furthermore, Janus kinase (JAK) inhibitors, such as ruxolitinib, mitigate inflammatory damage by suppressing cytokine production—including IFN-γ, TNF-α, IL-6, and IL-17—through inhibition of JAK1/2 signaling, further expanding the therapeutic landscape for GvHD management [135–137].

## Allorejection and strategies to address allorejection

In allogeneic cell-based immunotherapy, besides GvHD, allorejection also limits therapeutic efficacy. The host immune system recognizes allogeneic grafts as foreign and eliminates them, reducing the persistence of therapeutic cells. This occurs via direct allorecognition, where recipient T cells recognize donor HLA molecules, and indirect allorecognition, where donor antigens are processed by recipient APCs and presented to host CD4 + T cells, triggering immune responses [36].

To mitigate allorejection, genetic engineering of therapeutic cells is widely used. *B2M* (β2-microglobulin) KO disrupts HLA class I expression, while *CIITA* KO prevents HLA class II expression, reducing recognition by host T cells [36]. Therapies such as CTX110 and CTX130 use *B2M* KO, whereas TyU19 employs *CIITA* KO via CRISPR-Cas9, enhancing CAR-T cell persistence [70, 73, 80].

Other strategies involve partial HLA matching and stem cell-derived platforms like HSPCs and iPSCs, which provide a renewable source of cells engineered for reduced immunogenicity [36]. Immunosuppressive regimens also play a crucial role in preventing allorejection. Lymphodepletion, achieved through chemotherapy or monoclonal antibodies like alemtuzumab, creates a more favorable environment for engraftment [36]. To further enhance persistence, some allogeneic CAR-T therapies incorporate CD52 KO, allowing the therapeutic cells to evade alemtuzumab-mediated depletion. Notable examples of this approach include UCART19, ALLO-501, CTA101, ALLO-715, and UCART22 [66–69, 71, 138].

Clinical trials of allogeneic CAR-T therapies demonstrate promising outcomes. For example, UCART19 reported a median progression-free survival of 2.1 months and a median overall survival of 13.4 months, supporting the efficacy of these strategies [139].

## Clinical trial experiences using allogeneic cell products

Several clinical trials have been conducted to investigate the safety and efficacy of allogeneic cell therapies, focusing on minimizing GvHD and other safety concerns, such as dose-limiting toxicities (DLTs), while improving patient outcomes. These trials primarily use gene-editing strategies, including TCR disruption and *TRAC* CAR knock-in, and explore alternative cell sources with low GvHD risk, such as NK and NKT cells, to prevent GvHD while maintaining strong antitumor efficacy.

### Incidence of GvHD, other safety issues, and efficacy in TCR disruption approaches

One key strategy for preventing GvHD in allogeneic CAR-T cell therapies is disrupting the endogenous TCR through methods such as *TRAC* KO, *TRBC* KO, or *TRAC* CAR knock-in using TALENs or CRISPR-Cas9 [60, 64, 65]. For example, UCART19, an allogeneic CD19-targeting CAR-T therapy with *TRAC* knockout via TALENs, showed a low GvHD incidence (10% grade 1 acute skin GvHD) in both pediatric and adult B-ALL patients (NCT02808442, NCT02746952) [66]. Despite this, the therapy exhibited promising efficacy, with 67% of patients achieving CR and CAR-T cells persisting in 71% of patients for a median of 4.1 months [66]. However, the trial also observed other safety issues, including grade 1–2 CRS in 91% of patients, grade 3–4 CRS in 14%, prolonged cytopenia in 32%, and neurotoxicity in 38% [66]. These results underline the balance between efficacy and safety, where GvHD was manageable, but toxicities such as CRS and neurotoxicity remained concerning. Similar trials utilizing TALEN-based *TRAC* KO, including CD19-targeting ALLO-501 (NCT03939026), ALLO-501A (NCT04416984), and UCART19 (NCT02746952), BCMA-targeting ALLO-715 (NCT04093596), and CD22-targeting UCART22 (NCT04150497), reported either no GvHD or GvHD in a small fraction of patients (no more than 8% with grade 1 acute skin GvHD) [67–69, 138, 139]. Overall response rates (ORRs) ranged from 50 to 75%, with well-controlled adverse effects like DLTs and immune effector cell-associated neurotoxicity syndrome (ICANS) [67–69, 138, 139]. However, cytopenia was a common side effect, except for ALLO-715 and UCART22 [67–69, 138, 139].

CRISPR-Cas9-mediate *TRAC* KO therapies, including CD19-targeting CTX110 (NCT04035434) and TyU19 (NCT05859997), CD19/CD20-targeting CTA101 (NCT04227015), and CD7-targeting WU-CART007 (NCT04984356) targeting CD7, also effectively prevented GvHD [70, 72–74]. However, except for TyU19, these trials exhibited significant CRS in 56–83.3% patients, with low incidences of ICANS (9.4% in CTX110, 8.3% in WU-CART007) and no DLTs [70, 72–74]. Efficacy was favorable, with ORR reaching 67% (CTX110) and CR rates as high as 83.3% (CTA101), with persistent responses up to 86 days (WU-CART007) [70, 72–74].

*TRBC* KO approaches are less common in clinical trials due to the complexity of disrupting two constant regions. The only reported trial, P-BCMA-ALL01 (NCT04960579), employs Cas-CLOVER™ gene editing to eliminate TCR expression [74]. Similar to *TRAC* KO therapies, it prevents GvHD while maintaining a manageable safety profile, with 14% CRS (grade 1) and 4% ICANS, and no DLTs [74].

While *TRAC* CAR knock-in strategies avoid random CAR gene integration associated with lentiviral methods, they remain underutilized in clinical settings [76]. The two reported trials, CD70-targeting CTX130 (NCT04438083) and CD19-targeting TT52CAR19 (NCT04438083), reported no GvHD in CTX130 and a 16.7% incidence in TT52CAR19 [79, 80]. Both trials reported no DLTs or ICANS but exhibited notable CRS, affecting 50% of patients in CTX130 and 33.3% in TT52CAR19 [79, 80]. These findings highlight the effectiveness of gene-editing technologies in preventing GvHD and enhancing safety, though CRS and cytopenia remain significant concerns.

### Incidence of GvHD, safety issues, and efficacy in NK and NKT cell-based therapies

NK and NKT cells offer an alternative approach to allogeneic cell therapies with a reduced risk of GvHD due to their lower dependence on HLA recognition and reduced alloreactivity.

Early-phase trials of NK cell therapies, such as the HLA-mismatched CD19-targeting CAR-NK therapy (NCT03056339), reported no GvHD and demonstrated a favorable safety profile with no CRS or neurotoxicity [83]. Efficacy was notable, with a 73% response rate, and CAR-NK cells persisted at low levels for up to 12 months, supporting their potential as a safer alternative to TCR knockout CAR-T therapies while maintaining antitumor activity [83].

Similarly, NKT cell therapies, such as the allogeneic CD19-targeting CAR-NKT (NCT00840853) trial, showed promising results [18]. No GvHD or major safety concerns were reported, and grade 1 CRS occurred in 20% of patients [18]. The therapy demonstrated efficacy in ALL and non-Hodgkin lymphoma (NHL), with CR in one of two ALL patients and two of seven NHL patients [18]. NKT cells persisted for up to 12 weeks post-infusion, underscoring their potential for allogeneic applications [18]. However, further studies are needed to assess long-term durability and potential risks, such as cytopenia.

### Comparison of TCR knockout and NK/NKT cell-based therapies

TCR knockout and NK/NKT-based therapies take distinct approaches to reducing GvHD. TCR knockout strategies, as seen in UCART19 and ALLO-501, employ gene editing to eliminate endogenous TCRs, preventing GvHD while maintaining CAR-T efficacy [66, 67]. These approaches have demonstrated high ORR and CR rates in clinical trials. However, challenges such as CRS, neurotoxicity, and cytopenia remain.

In contrast, NK and NKT cell therapies inherently avoid TCR-mediated GvHD by relying on cells with minimal MHC dependence and low alloreactivity [47, 140]. These therapies have demonstrated superior safety profiles in early trials, with fewer CRS or neurotoxicity cases and no reported GvHD [18, 83]. Their efficacy is also promising, with NK cells persisting for up to 12 months and NKT cells for up to 12 weeks post-infusion [18, 83]. This suggests that NK and NKT-based approaches could offer a viable, safer alternative to TCR knockout CAR-T therapies while maintaining therapeutic effectiveness.

### Challenges in translating preclinical findings into clinical success

Despite promising preclinical results, translating these therapies into successful clinical treatments remains challenging. One major issue is immune regulation—while preclinical models often demonstrate robust responses to CAR-engineered therapies, human patients exhibit more variable immune reactions, increasing the risk of adverse effects [141, 142]. CRS and neurotoxicity, which are not always observed in animal models, have become prominent challenges in clinical trials [143–145]. Furthermore, although gene-editing strategies like TCR knockout effectively mitigate GvHD, concerns remain regarding the long-term persistence of CAR-T cells and their ability to sustain tumor targeting without off-target effects [2].

Cell exhaustion is another significant obstacle. In CAR-T cell therapy, multiple infusions or high doses can exacerbate cytopenia and worsen the risk of adverse events [146]. Therefore, improved strategies are needed to regulate immune responses, minimizing severe side effects such as CRS and neurotoxicity while ensuring the sustained persistence and efficacy of CAR-T or NK cells in targeting tumors.

## Manufacturing considerations for allogeneic cell products

Manufacturing allogeneic cell products presents unique challenges due to the high cell dose requirements, need for cost efficiency, and stringent quality control necessary for clinical and commercial applications. Robust manufacturing strategies are essential to achieve scalable production while ensuring product effectiveness and safety to make allogeneic cell products viable for clinical applications.

Many allogeneic therapies require cell doses exceeding 10^9^ cells, prompting the adoption of suspension-based manufacturing approaches, preferably without microcarriers, to minimize costs, simplify workflows, and avoid the disadvantages associated with particulate disadvantages [147]. One commonly used tool is the stirred tank bioreactor (STR) which has well-characterized scale-up transfer kinetics [36, 148]. Since STRs are closed, automated, and compatible with Good Manufacturing Practice (GMP) co-based culture systems featuring an in-process control, T cell manufacturing in STRs significantly reduces labor demands, batch-to-batch variation, and the risk of contamination [149, 150]. However, large-scale expansion platforms like STRs face limitations such as shear forces, impeller impact, uneven gas exchange, varying hydrostatic pressures, and an overall nonphysiological environment, all of which can impact the quality of the final product [149, 150]. These challenges highlight the need for further optimization to make STRs more suitable for clinical applications.

One critical step in the production of allogeneic CAR-engineered cells is genetic editing, which typically involves knocking out TCR and MHC-I/II alleles to enhance safety. However, though current gene-editing technologies including ZFNs and MegaTALs, CRISPR-Cas9, and TALENs allow successful genetic modifications, issues with inefficiency and off-target effects persist [148]. These limitations can potentially result in fatal GvHD, compromising the safety of the final allogeneic cell products [151]. Given the risk associated with genetic editing, implementing a standardized quality control process is essential to ensure the safety and efficacy of cell products, which further complicates the achievement of desired cell yield and cost efficiency.

Another challenge related to the production of allogeneic CAR-engineered cells is the technology transfer and process validation for each new manufacturing site [152]. Currently, there are two ways to conduct product comparability testing. First, batches of allogeneic CAR-engineered cell products are compared with prespecified and validated acceptance criteria, such as percent transduction and so on, which requires comprehensive knowledge of the product to establish meaningful acceptance criteria [153]. The other approach is to compare CAR-engineered cells produced from the same starting cell population [153]. Besides testing the comparability, all release assays and quality-control equipment at each manufacturing site must be tested and validated, further complicating the distribution of allogeneic CAR-engineered cell manufacturing [152].

Although achieving high cell doses, cost efficiency, safety, and effectiveness simultaneously remains challenging, the manufacturing of allogeneic CAR-engineered cells holds significant promise. For example, off-the-shelf CAR-T therapies offer the potential for streamlined production by generating multiple cryopreserved batches of cells from a single healthy donor, which not only enhances manufacturing efficiency but also reduces costs for both healthcare institutions and patients [154]. Overall, significant advancements and innovations in manufacturing techniques are still required to further simplify the production process and ensure these therapies are accessible, cost-effective, and scalable for widespread clinical use.

## Conclusions and perspectives

Allogeneic CAR-engineered cell therapies hold immense potential in broadening access to transformative immunotherapies for cancer patients. However, the risk of GvHD remains a major immunological challenge, requiring innovative strategies that balance therapeutic efficacy with safety. Clinically, GvHD manifests as severe immune-mediated damage, predominantly affecting the gastrointestinal tract, skin, and liver, and in rare cases, can lead to life-threatening complications [29, 30]. Addressing this risk is critical for the successful translation of allogeneic cell-based therapies into widely accessible treatments.

Current efforts to enhance the safety and efficacy of allogeneic cell therapies focus on eliminating the endogenous TCR or employing alternative immune cell sources. While HLA matching can reduce alloreactivity, it is constrained by donor availability, time limitations, and cost, making it impractical for large-scale application. More refined strategies include genome-editing approaches to prevent GvHD while preserving cytotoxic function. Technologies such as CRISPR-Cas9, TALENs, and ZFNs enable precise disruption of the *TRAC* and *TRBC* genes, thereby eliminating αβ TCR expression [58–62]. This approach has been validated in CAR-T cells targeting T-ALL and lymphoma, demonstrating reduced alloreactivity without compromising tumor clearance [65]. A complementary strategy involves integrating the CAR construct into the *TRAC* locus, ensuring uniform CAR expression while simultaneously preventing endogenous TCR activation.

Beyond T cells, alternative immune cell types—including NK cells, γδ T cells, and NKT cells—offer promising allogeneic platforms, as their TCRs are not MHC-restricted, thereby mitigating the risk of GvHD [82, 92, 100]. Additionally, Tregs and MSCs have been explored for their immunosuppressive properties, potentially modulating inflammatory responses and limiting T cell-mediated toxicity [123, 124]. Another emerging strategy involves engineering inhibitory receptors to attenuate aberrant immune activation, enhancing the safety profile of allogeneic therapies. iPSCs present a scalable alternative, enabling the generation of engineered CAR-T cells with standardized characteristics [110]. However, TCR-deficient iPSC-derived T cells may be susceptible to NK cell-mediated rejection due to the absence of self-MHC molecules, necessitating the integration of NK-inhibitory ligands to counteract host rejection mechanisms [36].

Clinical trials evaluating these approaches continue to show promising results. Gene-editing strategies such as *TRAC* knockouts and *TRAC* CAR knock-ins have been successfully implemented in trials, including UCART19, ALLO-501, and CTX110, demonstrating reduced or absent GvHD with high response rates [67, 70, 139]. Alternative cell-based therapies, such as NK and NKT cell platforms, have also exhibited safety profiles in trials like NCT03056339 and NCT00840853, further supporting their clinical feasibility. Nevertheless, challenges persist, including immune rejection, cell exhaustion, and toxicity-related adverse events such as CRS and neurotoxicity [18, 83].

As allogeneic CAR-engineered cell therapies advance toward clinical translation, mitigating GvHD while preserving therapeutic efficacy remains a critical challenge. Gene-editing technologies, novel immune cell platforms, and engineered inhibitory receptors offer promising avenues to minimize alloreactivity and enhance treatment safety. While early clinical trials demonstrate encouraging outcomes, challenges such as immune rejection, cell exhaustion, and inflammatory toxicities necessitate further refinement. Continued innovation in cellular engineering, coupled with a deeper understanding of immune regulation, will be essential in establishing allogeneic CAR therapies as a scalable and accessible immunotherapy for cancer patients worldwide.

## Data Availability

No datasets were generated or analysed during the current study.

## Abbreviations

AAV: Adeno-associated virus
APC: Antigen presenting cell
BLI: Bioluminescence imaging
CAR: Chimeric antigen receptor
CAR19-DNT: CAR19-modified double-negative T cells
CAR19-Tconv: CAR19-modified conventional T cells
CCL: Chemokine (C–C motif) ligand
CNI: Calcineurin inhibitors
CR: Complete response
CRS: Cytokine release syndrome
CXCL: Chemokine (C-X-C motif) ligand
DC: Dendritic cell
DLTs: Dose-limiting toxicities
FasL: Fas/Fas ligand
GI: Gastrointestinal
GMP: Good manufacturing practice
GvHD: Graft-versus-host disease
HDR: Homology-directed recombination
HLA: Human leukocyte antigen
HvG: Host-versus-graft
IDO: Indoleamine 2,3-dioxygenase
IFN: Interferon
IL: Interleukin
iPSC: Induced pluripotent stem cell
KO: Knockout
LPS: Lipopolysaccharides
MHC: Major histocompatibility complex
MLR: Mixed lymphocyte reaction
MSC: Mesenchymal stem cell
NCG: NOD/ShiLtJGpt-Prkdcem26Cd52Il2rgem26Cd22/Gpt
NFAT: Nuclear factor of activated T
NHL: Non-Hodgkin lymphoma
NK: Natural killer
NKT: Natural killer T
NOG: NOD.Cg-PrkdcscidIl2rgtm1Sug
NSG: NOD/SCID/IL2Rγnull
NT-DNT: Non-transduced double-negative T cells
PBMC: Peripheral blood mononuclear cell
PTCy: Post-transplant cyclophosphamide
STR: Stirred tank bioreactor
T-ALL: T-cell acute lymphoblastic leukemia
TALENs: Transcription activator-like effector nucleases
TCR: T cell receptor
TGF: Transforming growth factor
Th1: T-helper 1
Th2: T-helper 2
TME: Tumor microenvironment
TNF: Tumor necrosis factor
TRAC: T cell receptor alpha constant
TRBC: T cell receptor beta constant
Treg: Regulatory T cell
ZFNs: Zinc finger nucleases
